# Supplementary material for: Episodic events are flexibly encoded in both integrated and separated neural representations
Source: Nat Commun. 2026 Jan 19;17:752. doi: 10.1038/s41467-026-68473-6 (PMC12820042; doi:10.1038/s41467-026-68473-6)
Supplement: Supplementary file 1 — Supplementary Information [file 41467_2026_68473_MOESM1_ESM.pdf]

Supplementary Material

Episodic events are flexibly encoded in both integrated and separated neural representations

Zhenghao Liu[1*], Mikael Johansson[1] and Inês Bramão[1*]

[1]Department of Psychology, Lund University, Lund, Sweden

Supplementary Note 1: Episodic Detail Memory Tests

At the end of the memory task, participants completed a surprise episodic detail memory test, assessing their memory for both context and the clothing of the characters A and C. For the context memory test, participants were first shown an image of the Sim for 1.5 s, followed by the presentation of four possible context pictures, arranged in a 2×2 matrix: 1) the original context of the Sim, 2) the context of Sim with altered details, 3) the context of the corresponding, indirectly associated Sim, and 4) the context of the corresponding, indirectly associated Sim with altered details. Participants were asked to select the context in which the Sim originally appeared (see Figure S1A). Following the context memory test, participants were asked to retrieve the color and pattern of the Sim's clothing by choosing from four options (see Figure S1B). Participants rated their confidence on a three-point scale for all episodic detail memory tests: 1 – guessing, 2 – maybe, 3 – sure. There was no time limit for the responses, but responses made after 10s or associated with the confidence level 'guessing' were considered as incorrect response. For the current analyses, we included only trials where AB or BC associations were correctly retrieved, ensuring that surprise test results were based on accurate associative memory performance.

Memory for context was evaluated in two steps. First, we tested if participants incorrectly attributed the context of a Sim to its corresponding Sim due to memory integration. Previous studies suggest that, after forming an indirect AC association, participants may mistakenly recall the Sims' context as belonging to its corresponding Sim[1]. To this end, we ignored altered details and considered all trials correct for which participants selected the context of the current Sim (original or with altered details) and all trials incorrect for which the context of the corresponding Sim was selected (original or with altered details). A linear mixed-effects model tested if context memory varied as a function of AC association accuracy. The results showed that the effect of AC accuracy was not significant ($F(1, 32) = 2.110$, $p = 0.156$,

η_p^2 = 0.06, 95% CI = [0.00, 1.00]). Thus, we found no statistical evidence that participants were more likely to select the context of the corresponding Sim when the AC association was correctly retrieved than when it was not.

Next, we tested whether participants retained the fine-grained contextual details following memory integration. We considered all trials correct for which participants selected the original, unaltered context and all trials incorrect for which participants selected the context with altered details. We separately analyzed trials for which participants selected the contexts of the current Sim and trials for which participants selected the contexts of the corresponding Sim. Using linear mixed-effects models, we contrasted context memory as a function of AC accuracy (Correct vs. Incorrect). The results (Figure S1C) showed that when participants selected the contexts of the current Sim, contextual detail memory performance was not significantly affected by AC accuracy (F(1, 809) = 0.272, p = 0.602, η_p^2 = 3.36e-4, 95% CI = [0.00, 1.00]). Similarly, when participants selected the two contexts of the corresponding Sim, the effect of AC accuracy was also non-significant (F(1, 119) = 2.663, p = 0.105, η_p^2 = 0.02, 95% CI = [0.00, 1.00]).

For the clothing memory test, we examined whether its accuracy and confidence vary as a function of AC Accuracy. A marginal effect of AC Accuracy was found for clothing memory accuracy (F(1, 1422) = 3.698, p = 0.055, η_p^2 = 2.59e-3, 95% CI = [0.00, 1.00]), but not for confidence (F(1, 775) = 0.805, p = 0.370, η_p^2 = 1.04e-3, 95% CI = [0.00, 1.00]). Numerically, detail memory for clothing was worse after making correct AC inference (accuracy = 0.556) compared with an incorrect AC inference (accuracy = 0.612), although this difference was not statistically significant (Figure S1D).

In sum, unlike source memory, which showed a clear link to AC retrieval, no statistical evidence suggested that AC association performance systematically affects episodic memory details for context and clothing in the present study.

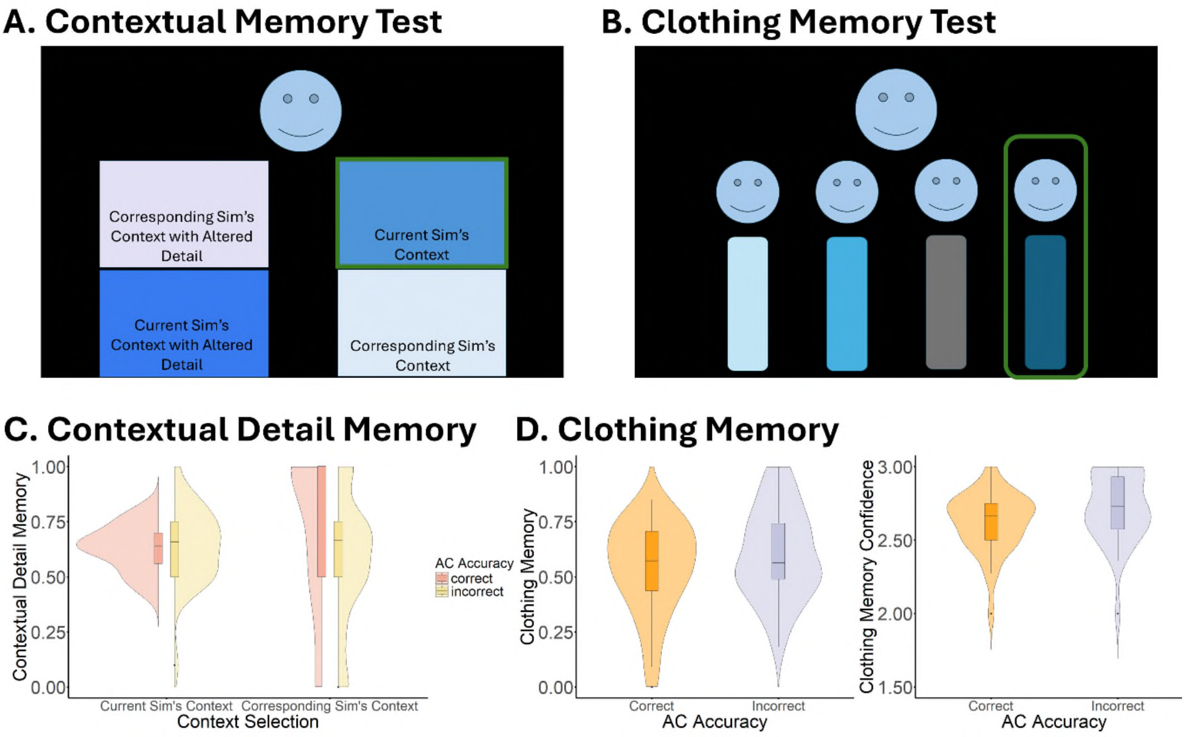

Figure S1. Surprise memory tests for episodic details and summary of the results. Experimental stimuli were created using The Sims 4 (© Electronic Arts Inc.). Placeholders are used for illustrative purposes only. (A) Context memory was tested by asking participants to select the context associated with a given Sim from four different alternatives: 1) the current Sim's original context, 2) the current Sim's context with altered details, 3) the corresponding Sim's original context, and 4) the corresponding Sim's context with altered details. (B) Clothing memory was tested by asking participants to select the correct clothes of a given Sim. (C) Contextual detail memory accuracy: Proportion of trials where participants selected the original context over the altered version. Data is grouped by current Sim's context vs. corresponding Sim's context and further split by AC association accuracy. Raincloud plots show the distribution of the data, with accompanying box plots indicate summary statistics. The mean is shown by the central bar, and the box captures the middle quartiles (25th-75th percentile). Whiskers reflect 1.5 interquartile range, and any values outside this span are displayed as dots. (D) Clothing memory accuracy and confidence as a function of indirect AC association accuracy. Violin plots show the sample distribution, with box plots overlaid. The central line of the box plot indicates the mean, the box represents the interquartile range (25th-75th percentile), and whiskers extend to 1.5 inter quantile range. Individual data points beyond the whiskers are plotted as dots. Plots were generated using data from 36 participants in a repeated-measures design.

Supplementary Note 2: Models for Assessing Behavioral Performance

To assess performance on the associative memory tests and on the source memory test, we ran linear mixed models on accuracy, and response times and confidence of correct responses. For all statistical models, and to prevent model overfitting[2,3], each model started with the least complexity, i.e., with participants as the single random intercept. The inclusion of other effects as random slopes was evaluated by looking at the model fitting. If the model fitting improved with the inclusion of a given random slope, the random slope was kept. The final equations for each model are displayed in Table S1.

Table S1. Mixed linear models used for behavioral performance assessment.

	Equation
Associative Memory	
	$\text{accuracy} \sim \text{Association Type} + \text{intercept} + (\text{intercept}\mid\text{participant})$
	$\text{response times} \sim \text{Association Type} + \text{intercept} + (\text{intercept}\mid\text{participant})$
	$\text{confidence} \sim \text{Association Type} + \text{intercept} + (\text{intercept}\mid\text{participant})$
Source Memory	
	$\text{accuracy} \sim \text{Association Type} + \text{Association Accuracy} + \text{Association Type} * \text{Association Accuracy} + \text{intercept} + (\text{Association Type} + \text{Association Accuracy} + \text{Association Type} * \text{Association Accuracy} + \text{intercept}\mid\text{participant})$
	$\text{confidence} \sim \text{Association Type} + \text{Association Accuracy} + \text{Association Type} * \text{Association Accuracy} + \text{intercept} + (\text{Association Type} + \text{intercept}\mid\text{participant})$

Supplementary Note 3: Representational Similarity Within and Between AB Movies

Before investigating neural similarity between AB and BC movies, we first examined representational similarity within and between AB movies. Using the EEG data recorded during AB movie encoding, which included repeated presentations of the same AB movie, we compared neural similarity across repetitions of the same AB movie against that of different AB movies. This analysis served as a validation step to assess whether our methodological approach could reliably capture neural pattern similarity driven by shared visual input.

Specifically, we extracted the feature vectors for each AB movie segment across different repetitions and correlated them with the feature vector at each timepoint of the entire epoch of the same AB movie (in other repetitions) as well as different AB movies. This procedure followed the same time-resolved analysis pipeline used for the AB-BC similarity analyses (see Methods in the main text).

As shown in Figure S2, the results confirm that our method captured common visual input in two complementary ways: (1) within-segment similarity: Neural similarity was higher across repetitions of the same segment than across different AB movies, reflecting consistent responses to identical visual input; and (2) generalization driven by visual overlap: *Sim A* showed similarity to 'Sim A in Context' and to 'Sim A& B in Context'; 'Sim A in Context' showed similarity to 'Sim A&B in Context'; and 'Sim B' showed similarity to the 'Sim A in B in Context'.

Together, these findings validate that our methodological pipeline successfully captures representational similarities driven by perceptual input, providing a prerequisite for investigating memory-related neural similarities.

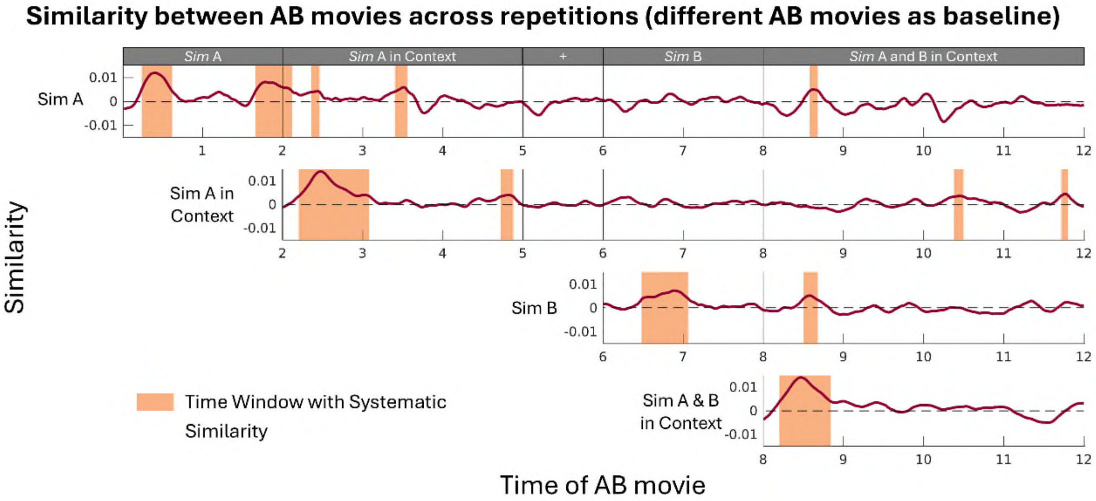

Figure S2. Neural representational similarity differences between repetitions of the same AB movie and different AB movies. Time windows with systematic similarities are indicated by orange shading.

Supplementary Note 4: Topographic Distribution of the Representational Similarity Effects

To explore the topographical distribution of the representational similarity effects, we ran our time-resolved representational similarity analysis pipeline with one channel and its immediate neighbors at a time (for a similar procedure, see references[4,5]). This yielded a time course of the neural pattern similarity between AB and BC movies at each channel, allowing us to reconstruct the topographical distribution of neural pattern similarities between AB and BC movies across the time windows that showed significant effects (see Figure 5B).

The results are summarized in Figure S3. Both similarity and dissimilarity effects were broadly distributed across the scalp without a clear topography. Interestingly, this widespread topography overlaps with the findings from the univariate analysis (see Figure 6). This suggests that while these processes may originate from distinct hippocampal subregions, they likely engage widespread cortical networks and reflect large-scale neural activity.

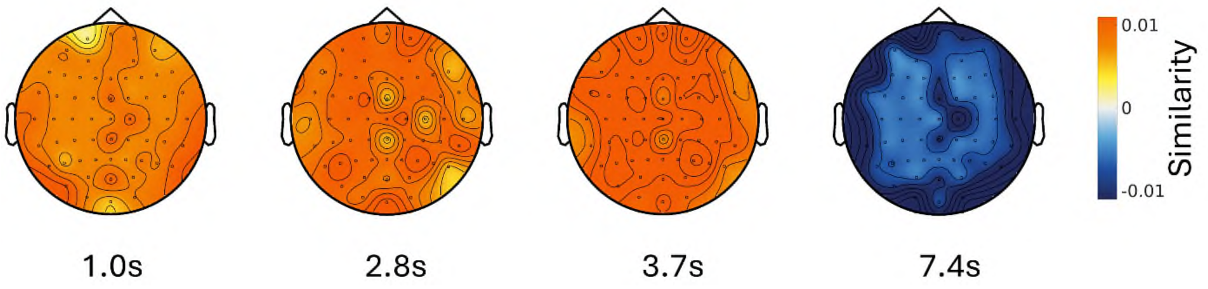

Figure S3. Topographical distribution of similarity and dissimilarity between 'Sim A&B in Context' segment and BC movie. Colors represent differences in Pearson correlation (*r*) values between AB and the corresponding BC movie versus AB and the non-corresponding BC movie.

Supplementary Note 5: Representational Similarity Across the Five Repetitions of Each Movie

Here, we show how the neural similarities and dissimilarity evolve across the five encoding repetitions of the BC movie (Figure S4). For each participant, movie, and repetition, the neural (dis-)similarity values were extracted for the time windows with significant results in the main analysis (see Figure 4).

First, we tested if these neural (dis-)similarities were already present in the initial encoding of the BC movie by contrasting the patterns of the initial repetition against zero. This hypothesis was not supported by the data (maximum BF_{10} = 0.998), providing anecdotal evidence for the null hypothesis, with no similarities or dissimilarities observed during the initial repetition. This is not surprising because, during the first repetition, participants would not yet know the correspondence between AB and BC movies until Sim B was presented.

Next, we tested whether a linear trend emerged for neural (dis-)similarities across the five repetitions. A Bayesian linear regression was applied to predict the neural (dis-)similarities throughout the five rounds of encoding. The results showed that the similarities observed at 1.0 second followed such a trend during the first three encoding rounds (β = 0.138, BF_{10} = 3.789). The linear progressive pattern was also observed for the similarity observed at 3.7 seconds (β = 0.125, BF_{10} = 5.291) across the five encoding rounds. However, no statistical evidence for such linear trend was found for the similarity observed 2.8 seconds (maximum BF_{10} = 1.693). The dissimilarity observed at 7.4 seconds (β = -0.157, BF_{10} = 8.809) showed a linear trend for the first four rounds.

These findings suggest that neural similarities and dissimilarities gradually shift over the five encoding repetitions. The observed linear trends in similarity and dissimilarity likely indicate an iterative adaptation of the integrated and separated representations over time, as the brain progressively refines the memory encoding process with each repetition.

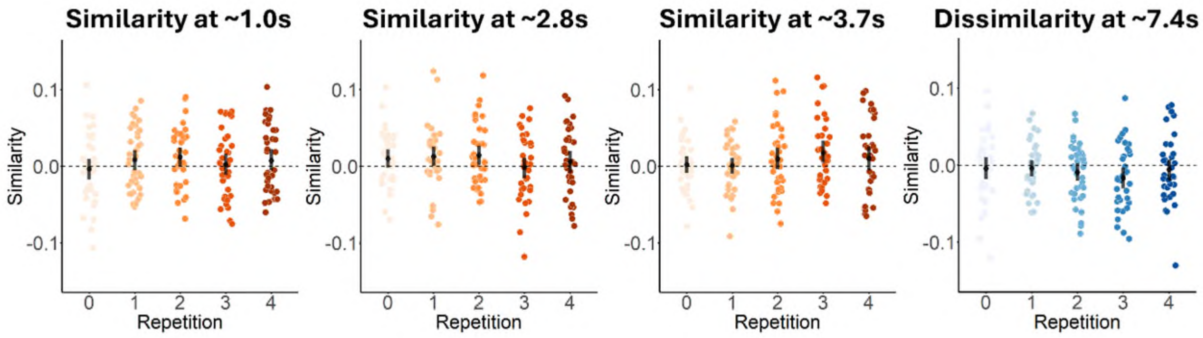

Figure S4. Progression of similarity and dissimilarity across the five encoding repetitions of the BC movie. Error bars indicate the 95% confidence interval for each repetition, estimated via bootstrapping. Each dot represents an individual participant (N = 36). Similarities are indicated in orange and the dissimilarity in blue, the depths of which refer to different repetitions.

Supplementary Note 6: Correlation between Neural Similarities and Dissimilarity

To test for a trade-off between integration and separation, we calculated partial correlations between observed similarities and dissimilarities at the trial level, controlling for repetition effects. This measure captures their co-occurrence within a single trial, providing a basis for inferring such a potential trade-off. The partial correlation matrix between the observed similarities and the dissimilarity is summarized in Table S2, *p* values for two-tailed inferential statistics are also included. The results showed that the observed similarities tend to cooccur within the same trial, however, how their correlations with the dissimilarity were nonsignificant.

Table S2. Partial correlation coefficients (*r*) between the similarities and the dissimilarity across 36 participants. Each *r* value is followed by its corresponding two-tailed *p* value in parentheses. Significant correlations are indicated in bold ($p < 0.05$).

	Similarity at 1.0s	Similarity at 2.8s	Similarity at 3.7s
Similarity at 2.8s	0.001 (*p* = 0.950)		
Similarity at 3.7s	0.038 (*p* = **0.031**)	0.046 (*p* = **0.009**)	
Dissimilarity at 7.4s	0.021 (*p* = 0.230)	-0.016 (*p* = 0.357)	0.019 (*p* = 0.290)

Supplementary Note 7: Representational Similarity Analysis between Other AB Segments and Corresponding BC Movies

Beyond assessing the representational similarity between the segment corresponding to when Sim A and B interacted in a Context and the corresponding BC movie, we also estimated the similarity between other segments of AB movie and their corresponding BC movie. This analysis aimed to provide a more comprehensive view of how different phases of AB encoding relate to subsequent BC representations. The temporal dynamics of these similarities are plotted in Figure S5, highlighting fluctuations in the neural representational across different time windows. The specific time windows of systematic similarities, along with their statistics, are summarized in Table S3.

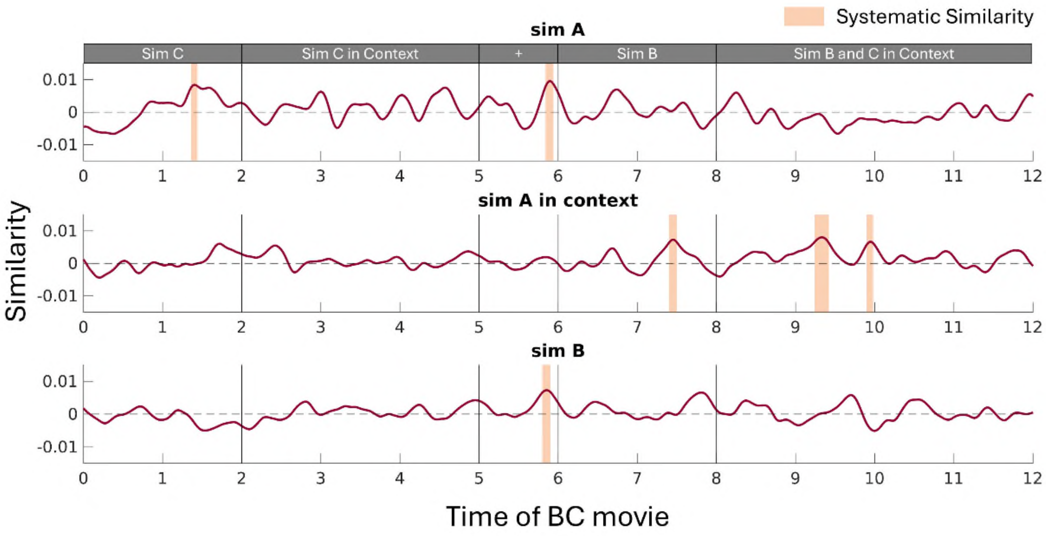

Figure S5. Neural representational similarities and dissimilarities between other AB movie segments and BC movie encoding. The times with systematic similarities are marked in highlighted and the statistics are summarized in Table S3.

Table S3. Summary of the time windows and statistics between other segments of AB movie and BC movie encoding.

	Time	Average Similarity	$BF_{10}s >$
Sim A			
	1.4s	8.4e-3	3.063
	5.9s	9.3e-3	3.160
Sim A in Context			
	7.5s	7.1e-3	3.099
	9.3s	7.5e-3	3.410
	10.0s	6.6e-3	3.003
Sim B			
	5.9s	7.2e-3	3.408

Supplementary Note 8: Relationship between Neural Pattern Similarities / Dissimilarities and Behavioral Memory Tests

Here we report the overall relationships between all the systematic (dis-)similarities and the behavioral performance of the memory tests, including AC inference, source memory, and memory for clothing. Memory for clothing was also included in the present analysis because it likely covaries with AC memory performance (see Supplementary Note 1). Specifically, we estimated how the similarity values relate to later clothing memory, with the repetition order and AC inference accuracy included as controlled variables. The results are summarized in Table S4.

In addition to what is reported in the main text, we observed that higher neural pattern similarity between Sim A observed at 1.4 seconds during BC movie encoding was associated with later AC inference performance. This aligns with our main finding, showing that the neural pattern similarities reflect the formation of an integrated representation involving the elements encountered across different events, which can later be used to respond to the AC memory test. Additionally, the similarities between Sim A in Context and BC movie, observed at 9.3 seconds and 10.0 seconds, were negatively related to memory for clothing, indicating that effort for memory integration may lead to the loss of episodic details associated with the individual events.

Table S4**.** Bayesian Linear Regression-based analysis investigating the relationship between the representational (dis-)similarities and the behavioral memory tests. Significant effects are marked in bold.

Segment	Time	AC inference accuracy		Source memory accuracy		Clothing memory accuracy	
		β	BF_{10}	*β*	BF_{10}	*β*	BF_{10}
Similarity to 'Sim A'							
	1.4s	**0.049**	**26.268**	-0.014	1.283	-0.031	2.755
	5.9s	0.012	1.214	0.028	2.811	0.005	1.013
Similarity to 'Sim A in Context'							
	7.5s	-0.027	2.779	-0.003	1.004	0.013	1.168
	9.3s	-0.004	1.011	-0.011	1.154	**-0.033**	**3.021**
	10.0s	-0.006	1.046	0.001	0.992	**-0.044**	**7.756**
Similarity to 'Sim B'							
	5.9s	-0.002	0.996	0.013	1.238	0.005	1.016
Similarity to 'Sim A and B in Context'							
	1.0s	0.022	1.922	0.000	0.992	-0.013	1.169
	2.8s	**0.044**	**14.668**	0.022	1.820	0.025	1.931
	3.7s	0.000	0.992	**-0.036**	**5.345**	-0.004	1.009
	7.4s	0.012	1.222	**-0.041**	**8.681**	0.001	0.992
N of participant			30		28		36

Reference

1. Carpenter, A. C. & Schacter, D. L. Flexible retrieval: When true inferences produce false memories. *J. Exp. Psychol. Learn. Mem. Cogn.* **43**, 335–349 (2017).
2. Matuschek, H., Kliegl, R., Vasishth, S., Baayen, H. & Bates, D. Balancing Type I error and power in linear mixed models. *J. Mem. Lang.* **94**, 305–315 (2017).
3. Liu, Z., Johansson, M., Johansson, R. & Bramão, I. The effects of episodic context on memory integration. *Sci. Rep.* **14**, 30159 (2024).
4. Bramão, I. & Johansson, M. Neural Pattern Classification Tracks Transfer-Appropriate Processing in Episodic Memory. *eneuro* **5**, ENEURO.0251-18.2018 (2018).
5. Bramão, I., Liu, Z. & Johansson, M. Remembering the past affects new learning: The temporal dynamics of integrative encoding. *Neuropsychologia* **212**, 109148 (2025).
6. Calinski, T. & Harabasz, J. A dendrite method for cluster analysis. *Commun. Stat. - Theory Methods* **3**, 1–27 (1974).
