# Supplementary material for: Episodic events are flexibly encoded in both integrated and separated neural representations
Source: Nat Commun. 2026 Jan 19;17:752. doi: 10.1038/s41467-026-68473-6 (PMC12820042; doi:10.1038/s41467-026-68473-6)
Supplement: Supplementary file 2 — Reporting Summary [file 41467_2026_68473_MOESM2_ESM.pdf]

nature portfolio

Corresponding author(s): Zhenghao Liu (zhenghao.liu@psy.lu.se); Inês Bramão (ines.bramao@psy.lu.se)

Last updated by author(s): Nov 15, 2025

Reporting Summary

Nature Portfolio wishes to improve the reproducibility of the work that we publish. This form provides structure for consistency and transparency in reporting. For further information on Nature Portfolio policies, see our Editorial Policies and the Editorial Policy Checklist.

Statistics

For all statistical analyses, confirm that the following items are present in the figure legend, table legend, main text, or Methods section.

n/a	Confirmed	
☐	☒	The exact sample size (*n*) for each experimental group/condition, given as a discrete number and unit of measurement
☐	☒	A statement on whether measurements were taken from distinct samples or whether the same sample was measured repeatedly
☐	☒	The statistical test(s) used AND whether they are one- or two-sided<br>*Only common tests should be described solely by name; describe more complex techniques in the Methods section.*
☐	☒	A description of all covariates tested
☐	☒	A description of any assumptions or corrections, such as tests of normality and adjustment for multiple comparisons
☐	☒	A full description of the statistical parameters including central tendency (e.g. means) or other basic estimates (e.g. regression coefficient) AND variation (e.g. standard deviation) or associated estimates of uncertainty (e.g. confidence intervals)
☐	☒	For null hypothesis testing, the test statistic (e.g. *F*, *t*, *r*) with confidence intervals, effect sizes, degrees of freedom and *P* value noted<br>*Give P values as exact values whenever suitable.*
☐	☒	For Bayesian analysis, information on the choice of priors and Markov chain Monte Carlo settings
☐	☒	For hierarchical and complex designs, identification of the appropriate level for tests and full reporting of outcomes
☐	☒	Estimates of effect sizes (e.g. Cohen's *d*, Pearson's *r*), indicating how they were calculated

Our web collection on statistics for biologists contains articles on many of the points above.

Software and code

Policy information about availability of computer code

Data collection	PsychoPy (v2022.1.0) was used for stimuli presentation and response collection. Curry 7 was used for EEG data collection.
Data analysis	Behavioral data were analyzed in R (4.1.2) using the packages lme4 (1.1-34), emmeans (1.8.7), effectsize (0.8.9), and BayesFactor (0.9.12). EEG data were analyzed using MATLAB R2023b.

For manuscripts utilizing custom algorithms or software that are central to the research but not yet described in published literature, software must be made available to editors and reviewers. We strongly encourage code deposition in a community repository (e.g. GitHub). See the Nature Portfolio guidelines for submitting code & software for further information.

Data

Policy information about availability of data

All manuscripts must include a data availability statement. This statement should provide the following information, where applicable:

- Accession codes, unique identifiers, or web links for publicly available datasets
- A description of any restrictions on data availability
- For clinical datasets or third party data, please ensure that the statement adheres to our policy

The behavioral and EEG data presented in this study are available in the Zenodo database [https://doi.org/10.5281/zenodo.17612987]. To support reproducibility, we have shared preprocessed datasets, providing a straightforward starting point for analysis. The EEG raw data underlying this study is part of an ongoing project with additional analyses planned through 2028. The full, anonymized dataset will be deposited in Zenodo database and made publicly accessible in January 2028.

Research involving human participants, their data, or biological material

Policy information about studies with human participants or human data. See also policy information about sex, gender (identity/presentation), and sexual orientation and race, ethnicity and racism.

Reporting on sex and gender	The present study includes data from 36 participants (27 females, 9 males), based on self-reported gender at the time of informed consent. Gender was not considered in the study design, as the research focuses on fundamental aspects of human memory that are not generally considered to vary by gender.
Reporting on race, ethnicity, or other socially relevant groupings	Race, ethnicity or other socially relevant grouping was not assessed.
Population characteristics	The final sample consisted of 36 participants (27 female, 9 male).
Recruitment	Participants were recruited through social media posts and flyers distribution at the Lund University campus. This recruitment approach may introduce some self-selection bias, as individuals who chose to participate could differ in motivation and interest, compared with those who did not volunteer. There is a potential bias toward participants who are motivated to earn money through research participation. However, because compensation was provided in the form of a shopping voucher rather than cash, this reduces the likelihood of attracting participants primarily seeking monetary gain. Additionally, because the study focused on basic cognitive functions of memory integration and separation, influence of motivational differences is likely to be minimal.
Ethics oversight	As established by Swedish authorities and specified in the Swedish Act concerning the Ethical Review of Research involving Humans (2003:460), the present study does not require specific ethical review by the Swedish Ethical Review Authority due to the following reasons: (1) it does not deal with sensitive personal data, (2) it does not use methods that involve a physical intervention, (3) it does not use methods that pose a risk of mental or physical harm, (4) it does not study biological material taken from a living or dead human that can be traced back to that person. The Ethics Committee at the Department of Psychology, Lund University, has corroborated that the present research protocol follows the research ethics guidelines established by Swedish authorities.

Note that full information on the approval of the study protocol must also be provided in the manuscript.

Field-specific reporting

Please select the one below that is the best fit for your research. If you are not sure, read the appropriate sections before making your selection.

☐ Life sciences ☒ Behavioural & social sciences ☐ Ecological, evolutionary & environmental sciences

For a reference copy of the document with all sections, see nature.com/documents/nr-reporting-summary-flat.pdf

Behavioural & social sciences study design

All studies must disclose on these points even when the disclosure is negative.

Study description	The present study is a quantitative experimental study, using within-participant design to investigate human memory.
Research sample	A total of 41 participants were recruited, of whom 5 were excluded based on pre-established criteria. The final sample therefore consisted of 36 participants (27 female, 9 male; Age: M ± SD = 24.3 ± 2.94). Participants were generally associated with the Lund University community and are therefore be relatively homogeneous in terms of age and educational background. Nevertheless, the characteristics of our sample is typical for studies investigating the neural mechanisms of human memory.
Sampling strategy	Following previous studies (e.g., Cohn-Sheehy et al., 2021; Schlichting et al., 2015), we aimed for a sample size of 35 participants. A convenient sampling strategy was used. Participants were associated with the community of Lund University, including university students, their family and friends.
Data collection	Behavioral data was collected by PsychoPy. EEG was recorded using a SynAmps RT Neuroscan 64-channel amplifier (sampling rate 1kHz, bandwidth DC-3500Hz, 24-bit resolution, left mastoid reference) with 62 electrodes attached to an elastic cap (active electrode EasyCap). No one, other than the researcher and participant, was present during data collection. Researchers were not blind to the hypotheses during data collection, however, instructions of task were embedded in the experimental program and constant for all participants.
Timing	2022-4-22 to 2022-5-30
Data exclusions	Data from one participant was excluded for not having at least one incorrect AC association trial. Additionally, the data from another four participants were excluded due to poor EEG data quality and/or experimental programming errors. Data exclusion was based on pre-established criteria.
Non-participation	No participant dropped out.

Randomization	No experimental group was assigned, the present study used within-participant design.

Reporting for specific materials, systems and methods

We require information from authors about some types of materials, experimental systems and methods used in many studies. Here, indicate whether each material, system or method listed is relevant to your study. If you are not sure if a list item applies to your research, read the appropriate section before selecting a response.

Materials & experimental systems

n/a	Involved in the study
☒	☐ Antibodies
☒	☐ Eukaryotic cell lines
☒	☐ Palaeontology and archaeology
☒	☐ Animals and other organisms
☒	☐ Clinical data
☒	☐ Dual use research of concern
☒	☐ Plants

Methods

n/a	Involved in the study
☒	☐ ChIP-seq
☒	☐ Flow cytometry
☒	☐ MRI-based neuroimaging

Plants

Seed stocks	NA
Novel plant genotypes	NA
Authentication	NA
